# Paraplegia due to intradural cement leakage after vertebroplasty: a case report and literature review

**DOI:** 10.1186/s12891-021-04625-7

**Published:** 2021-08-28

**Authors:** In-Hwa Baek, Hyung-Youl Park, Ki-Won Kim, Tae-Yang Jang, Jun-Seok Lee

**Affiliations:** 1 Department of Orthopedic Surgery, Eunpyeong St. Mary’s Hospital, College of Medicine, The Catholic University of Korea, 1021, Tongil-Ro, Eunpyeong-gu, 03312 Seoul, Republic of Korea; 2 Department of Orthopaedic Surgery, Yeouido St. Mary’s Hospital, College of Medicine, The Catholic University of Korea, Seoul, Korea

**Keywords:** Paraplegia, Intradural, Cement leakage, Vertebroplasty, Complication

## Abstract

**Background:**

Vertebroplasty (VP) is considered an alternative therapy in an osteoporotic compression fracture that failed conservative treatment. However, cement leakage into the intradural space can cause catastrophic complications. To the best of our knowledge, intradural cement leakage following VP has been reported only in 7 cases. We report here a case of intradural cement leakage following VP with a literature review.

**Case presentation:**

An 84-year-old female with an L1 osteoporotic fracture underwent percutaneous VP at a local hospital. Immediately after the procedure, she complained of weakness, numbness, and pain in both legs, and her back pain aggravated. She was transferred to our hospital. The initial muscle power was grade 2 for the right leg and grade 4 for the left leg. Computed tomography (CT) scan showed intradural cement leakage from T10 to L2. Magnetic resonance imaging showed an intradural mass lesion. Although we performed total laminectomy with durotomy and removed intradural cement completely, the neurological deficit did not completely recover. The muscle power was grade 3 for the right leg and grade 4 for the left leg at the last follow-up.

**Conlcusions:**

If a neurological deficit is found after VP, a CT scan should be taken to confirm the pattern of cement leakage. In case of intradural cement leakage, surgical decompression should be recommended to improve neurological deficit. To prevent intradural cement leakage during the VP, the needle tip should not perforate the medial wall of the pedicle with appropriate viscosity of cement.

## Background

Vertebral compression fracture is the most common complication of osteoporosis [1–3]. Traditional treatment for osteoporotic compression fracture includes bed rest, analgesics, muscle relaxants, brace, and physical therapy [2–4]. The majority of patients respond favorably to conservative treatments [2, 3, 5]. However, some patients suffer from persistent back pain and immobility. For these painful osteoporotic compression fractures, vertebroplasty (VP) is considered an alternative therapy for pain relief and fracture stabilization [6–8].

Cement leakage from the vertebral body into adjacent structures is one of the most common complications [9]. Most cases of the cement leakage have no clinical significance [10, 11]. However, cement leakage into the spinal canal can cause serious complications such as paraplegia by compressing the spinal cord [11]. Moreover, cement leakage to intradural space can cause catastrophic complications, although very rare [1, 6, 10, 12–15]. We report here a case of intradural cement leakage following percutaneous VP with a literature review.

## Case presentation

An 84-year-old female with underlying hypertension, asthma, and cured breast cancer presented with low back pain and had no neurological deficit. She underwent percutaneous VP using polymethylmethacrylate under the diagnosis of L1 osteoporotic vertebral compression fracture at a local hospital. Bone mineral density showed osteoporosis, where total lumbar and femur neck were − 3.1 and − 2.0 in T-score, respectively. Immediately after the procedure, she complained of weakness, numbness, and pain in both legs. Even after the conservative treatment, she continued to suffer from persistent back pain and could not ambulate although cane ambulation was possible before VP.

She was transferred to our hospital three weeks later after VP. Neurologic examination revealed hypesthesia of the left leg and paraplegia below L2 level. The muscle power was grade 2 for the right leg and grade 4 for the left leg. Preoperative laboratory investigations revealed mildly elevated levels of erythrocyte sedimentation rate (ESR, 27 mm/hr, normal range: 0–20) with a normal limit of C-reactive protein (CRP, 1.84 mg/L, normal range: 0.1-5.0). Anemia with decreased hemoglobin (Hb, 11.0 g/dℓ, normal range: 12.0–16.0) and elevated alanine aminotransferase (ALT, 43 IU/L, normal range: 5–40) were also observed.

**Fig. 1:**
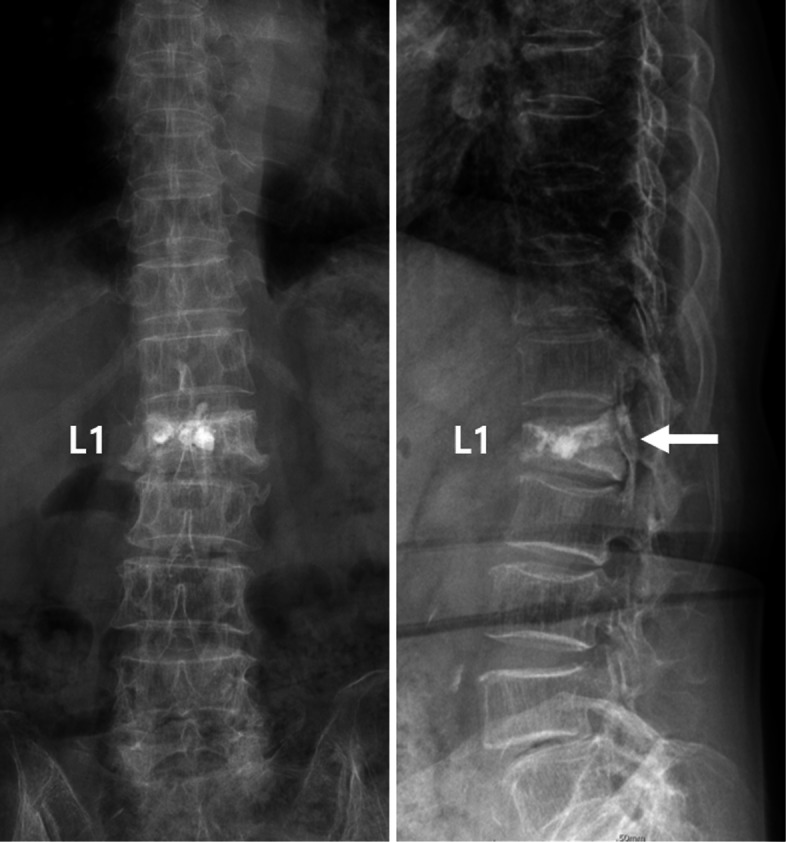
Anteroposterior and lateral plain radiographs of the thoracolumbar spine showing extravasation of the cement from the L1 vertebral body into the spinal canal

**Fig. 2:**
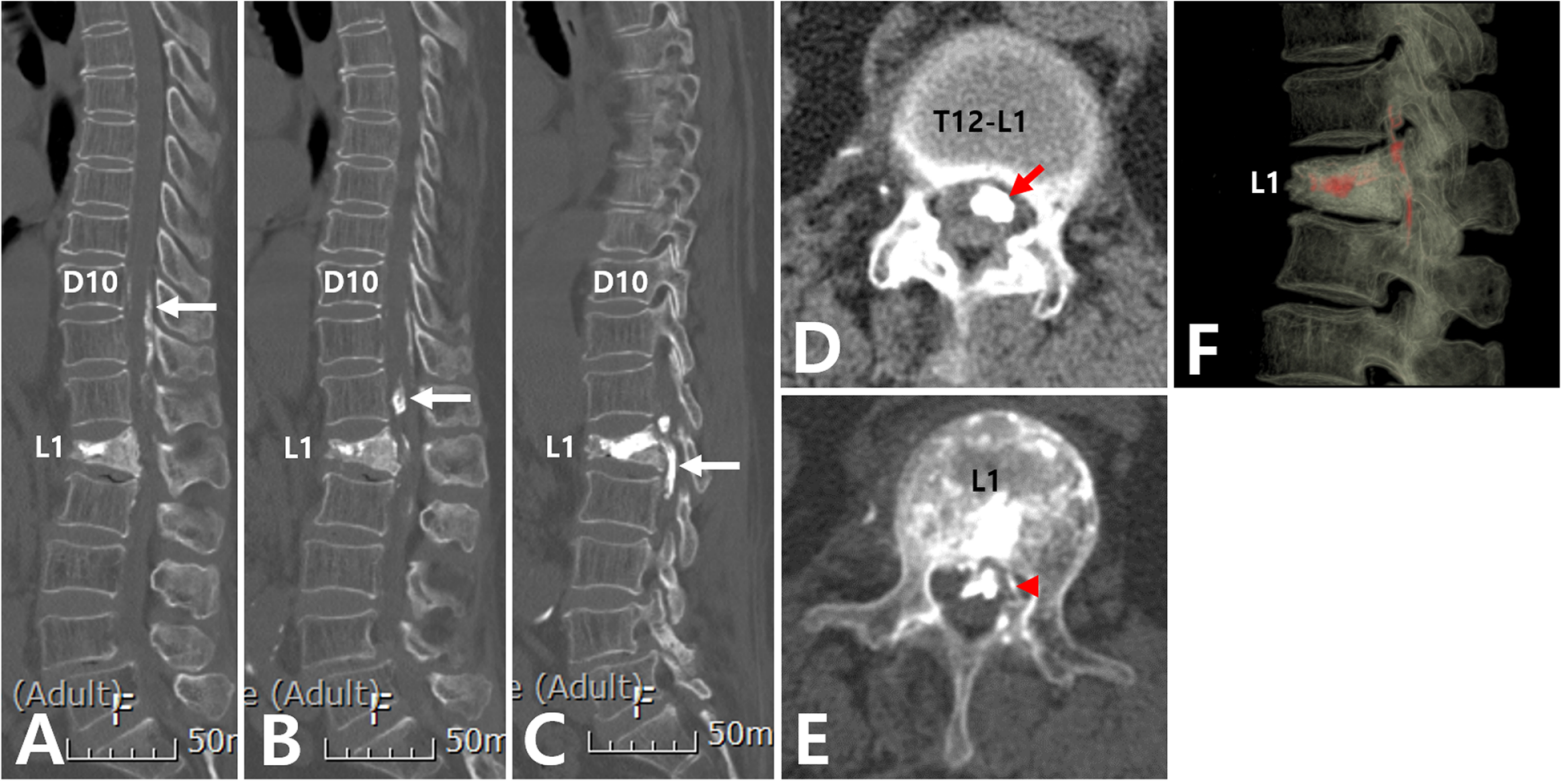
Computed tomography (CT) scans of the thoracolumbar spine. (**A, B, C**) A sagittal CT scan showing extravasation of the cement from T10 to L2 in the spinal canal. (**D**) Axial CT scan at the T12-L1 intervertebral disc level showing intradural cement leakage (arrow). (**E**) Axial CT scan at L1 vertebral body level showing cement leakage along a needle pathway from the medial to the left pedicle to the posterior wall of a vertebral body (arrowhead). (**F**) 3D CT reconstruction of the lumbar spine showing intradural cement leakage

**Fig. 3:**
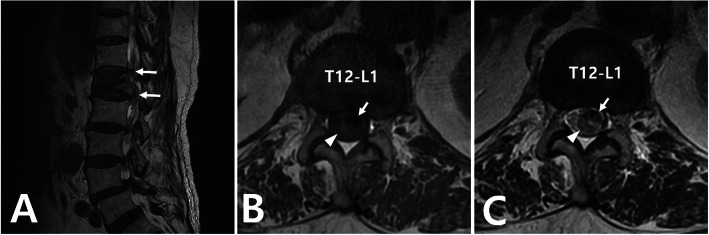
Magnetic resonance imaging (MRI) of the thoracolumbar spine. (**A**) Sagittal T2-weighted MRI showing cement leakage in the spinal canal (arrows). (**B**) Axial T1 and (**C**) T2-weighted MRI showing intradural cement leakage (arrows) with a displacement of the spinal cord to the right-posterior side (arrowhead)

Plain radiography of the thoracolumbar spine showed a compression fracture of the L1 with cement filling (Fig. 1). Extravasation of cement into the spinal canal was also found. Computed tomography (CT) scan showed intradural cement leakage from T10 to L2 (Fig. 2). Magnetic resonance imaging (MRI) also showed an intradural mass lesion that was hypointense on both T1-weighted and T2- weighted images (Fig. 3). Compression and displacement of the spinal cord to the right side secondary to the intradural mass lesion were observed.

**Fig. 4:**
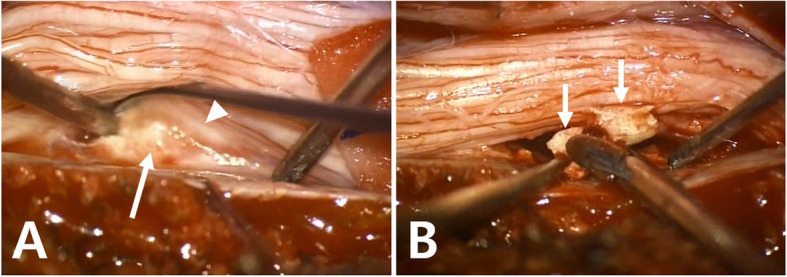
Intraoperative pictures after midline durotomy. (**A**) Adhesion between the spinal cord (arrowhead) and cement (arrow) was found. (**B**) Intradural cement (arrows) was removed piece by piece after it was broke down using a high-speed burr

Under the diagnosis of intradural cement leakage, emergent surgical decompression and cement removal was recommended to prevent the progress of neurological deficits. Surgical techniques and operative findings were as follows. First, stabilization by a posterior fixation from T10 to L3 was done. After that, the patient underwent total laminectomy and durotomy through a posterior approach from T12 to L2. Intradural cement was seen on the left-ventral side, compressing the cord to the right side (Fig. 4). Severe adhesion was also found between the spinal cord and the cement. We used a high-speed burr to prevent additional cord damage by breaking the cement with Kerrison punch or pituitary rongeur. The cement was removed piece by piece without additional cord injury. The durotomy was closed tightly after extensive irrigation. Pedicle screws were connected with rods and two cross-links.

**Fig. 5:**
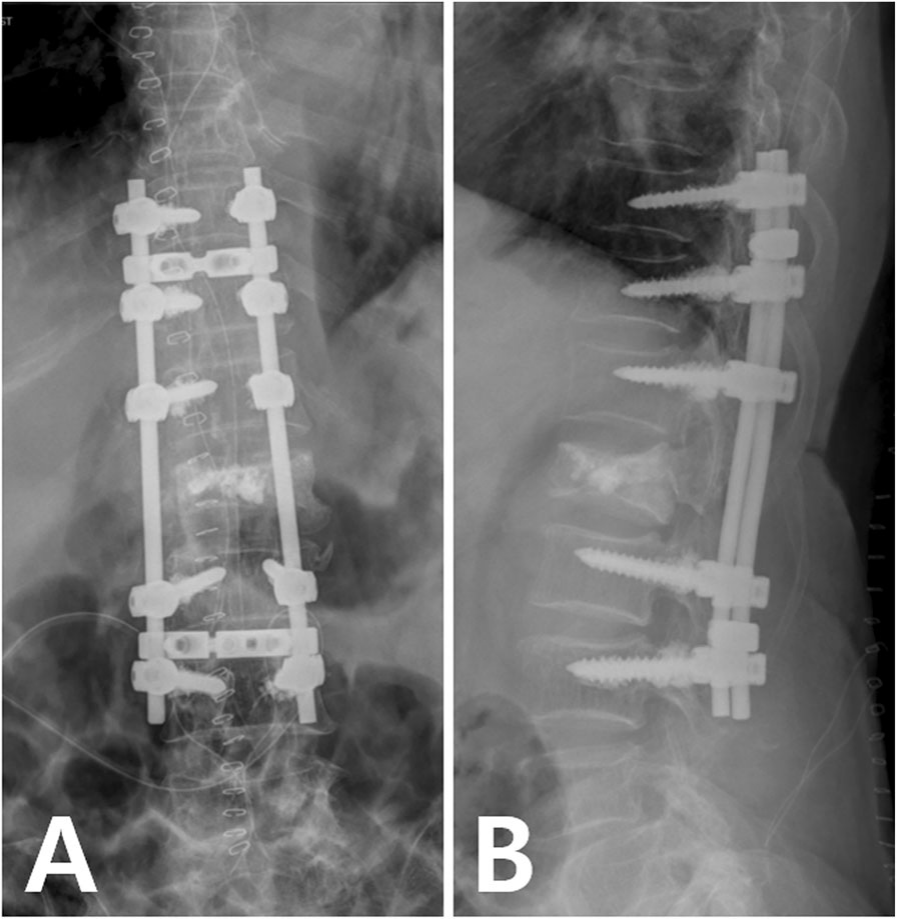
Anteroposterior and lateral plain radiographs of the thoracolumbar spine after surgery showing posterior fixation from T10 to L3. The cement in the spinal canal was removed completely

On postoperative 1 day, plain radiography showed successful removal of the intradural cement (Fig. 5). The leg and back pain were improved immediately after the surgery. Postoperatively, ESR and CRP were elevated up to 63 mm/hr (normal range: 0–20) and 49.67 mg/L (normal range: 0.1-5.0), respectively. Anemia and elevated ALT were also observed (Hb 9.4 g/dL and ALT 225 IU/L, respectively). The patient was referred to the department of rehabilitation medicine and discharged after 8 weeks. All laboratory values were within normal limits at discharge. Medications included a nonsteroidal anti-inflammatory drug (celecoxib), an opioid (tramadol) for pain control, and an antispasmodic agent (solifenacin) for neurogenic bladder. In addition, bisphosphonate (ibandronic acid) was injected for the osteoporosis postoperatively at 6 months. However, despite active rehabilitation for one year after the surgery, her muscle strength was minimally improved compared to preoperative muscle strength. The muscle power was grade 3 for the right leg and grade 4 for the left leg at the last follow-up. The patient has provided informed consent for the publication of this case report and accompanying images.

## Discussion and Conclusions

The first VP was performed in 1984 by French radiologists to treat a painful hemangioma of the cervical spine [5]. Since its introduction, the indication of VP has been extended to osteoporotic vertebral fracture, Kummell’s disease, and tumors such as metastatic disease, multiple myeloma, and painful aggressive hemangioma [9, 16]. Currently, the most common indication of VP is an osteoporotic vertebral compression fracture that has failed conservative treatment [9, 17]. Moreover, bone cement augmentation of the pedicle screws has been increasingly used to decrease the incidence of screw loosening in osteoporotic patients [18].

Reported complications of VP include infection, bleeding, transient radiculopathy, spinal stenosis, and pulmonary embolization [9]. However, most complications related to VP are minor and severe complications requiring additional treatment after VP have not reported in several randomized controlled trials [19–22]. Among complications of VP, extravasation of cement is relatively common. Hulme et al. [23] have reviewed 69 clinical studies and reported cement leakage in 41 % of VP. Similarly, Lee et al. [24] have reported cement leakage in 43 % of VP. Nonetheless, most cement leakages are asymptomatic or clinically insignificant [6, 25]. Lee et al. [24] have also reported symptomatic cement leakage in only 1.08 % of VP.

Regarding the route of cement leakage, systematic review reported 32.5 % in paravertebral space, 32 % in epidural space, 30.5 % into the disc, 3.3 % in foraminal space, and 1.7 % induced pulmonary emboli [23]. However, cement leakage into the intradural space after VP is very rare [11, 26]. To the best of our knowledge, it has been reported only in 7 cases (Table 1) [1, 6, 10, 12–15]. Above all, it is difficult to diagnose whether cement leakage is intradural or epidural by conventional radiography. Schmidt et al. [27] have reported that detection rates using conventional radiographs are low and complicated. The agreement rate between fluoroscopy/conventional radiographs and CT scan ranged only between 66 and 74 % [27]. Thus, it is necessary to confirm the feature of cement leakage with a CT scan after VP. In our case, we confirmed the accurate location of cement leakage on the CT scan.

**Table 1:** Intradural cement leakage during vertebroplasty reported in the literature

Author and year	Age	Sex	Fracture level	Type of disease	Operation	Time interval between surgery and VP	Lower extremity muscle power at initial (right/left)	Lower extremity muscle power at last follow-up (right/left)	Neurogenic bladder or bower
Shapiro et al. (2003) [10]	64	F	L2	OVCF	Laminectomy with durotomy and posterolateral fusion of L1-3	12 h	2/5	5/5	Not identified
Teng et al. (2006) [1]	79	F	L2	OVCF	Laminectomy with removal of intradural cement	Immediately	Not identified	Could not walk alone	Urinary incontinence, Constipation
Chen et al. (2006) [8]	90	F	T12, L1	OVCF	No surgery	No surgery	2/2	2/2	Not identified
Sabuncuoğlu et al. (2008) [11]	49	M	T12	Pathologic fracture d/t multiple myeloma	Laminectomy with durotomy	Not identified	1/5	4/5	Not identified
Kulkarni et al. (2013) [12]	48	F	L1	OVCF	1st op: Laminectomy of T12-L1 2nd op: Corpectomy of L1 3rd op: Durotomy	Not identified	1/2	3/4	Urinary incontinence
Lin et al. (2015) [4]	64	F	L2	OVCF	Laminectomy with durotomy	Not identified	3/5	4/5	Preserved anal tone
Grelat et al. (2018) [13]	64	M	L1	Pathologic fracture d/t malignant tumor	Laminectomy with durotomy and posterior short fixation	Not identified	2/5	Minimal improvement	Decreased anal tone

d/t = due to, F = female, M = male, op = operation, OVCF = osteoporotic vertebral compression fracture, VP = vertebroplasty

In terms of treatment, surgery for decompression and cement removal should be recommended for patients with intradural cement leakage. Because improvements in neurological deficits were observed in six patients undergoing surgery including present case [1, 6, 12–15]. However, Chen et al. [10] has reported that neurological deficit was sustained in one patient who refused open surgery because of old age. As surgical techniques, all patients underwent total laminectomy and durotomy with cement removal. When wide decompression is required or instability occurred, posterior fixation and fusion could be performed as in our case.

However, the optimal time of surgery has not been revealed. Shapiro et al. [12] have reported that one patient with motor weakness was recovered to normal at the last follow-up. They performed a laminectomy with posterior durotomy and removed intradural cement at 12 h after cement leakage. Although Teng et al. [1] have reported immediate surgery for intradural cement leakage, the patient could not walk alone. However, it might be due to insufficiently removed cement observed on follow-up MRI at 8 months after the surgery. In our case, the time interval between the VP and the surgery was about eight weeks. Our patient was unable to walk alone and the neurological deficit did not completely recover until the last follow-up. Her final lower extremity muscle power was grade 3/4 (right/left).

Meanwhile, thermal injury of intradural cement leakage to neural tissues should be considered. Lai et al. [28] has evaluated the peak temperature and duration above 45 °C at the posterior cortex and vertebral center in a porcrine model with cement leakage. Based on this result, they reported that the exothermic reaction at the posterior cortex might result in thermal injury to the neural tissue in case of cement leakage into the spinal canal. If thermal injury to neural tissues is critical, immediate decompression might be not necessarily needed. In this regard, further studies are needed to validate the optimal surgery time, including thermal injury to the neural tissue due to cement leaking into the intradural space. However, if the neurological deficit occurs and intradural cement leakage is confirmed on CT scan, decompressive surgery with cement removal does not have to be postponed.

What should we do to prevent intradural cement leakage? First, needle position is an important factor during VP. Intradural cement leakage can occur when the needle tip perforates the medial wall of the pedicle and passes through the dural in the spinal canal. Sabuncuoğlu et al. [13] have found a needle hole on the right side lamina at the VP level and another needle hole injury on the posterior dural area with cerebrospinal fluid leakage during operation. Shapiro et al. [12] have also reported cement leakage along the transdural needle path from the medial side of the left pedicle on axial CT scan. Teng et al. [1] have found that the needle has broken the medial wall of the pedicle and made a perforation of the dural sac on MRI. Similar to previous reports, we also found a cement leakage along a needle pathway from the medial side of the left pedicle to the posterior wall of a vertebral body and intradural cement on a CT scan (Fig. 2E). Authors think that intradural cement leakage can occur when the needle tip perforates the medial wall of the pedicle and passes through the dural in the spinal canal. Thus, during the procedure, the needle tip should not cross the medial border of the pedicle on the anteroposterior view before it reaches the posterior cortex of the vertebral body on the lateral view [6, 29].

Cement viscosity is another important factor [7, 23]. Viscosity has been proved to be a key influencing factor for leakage [30]. A paste-like consistency is suitable. Too low viscosity of cement can easily flow into the intradural space along the needle track. If an appropriate viscosity of cement is used, intradural cement leakage may not occur even in case of dural perforation of the needle. Moreover, the needle must be removed after confirming that the cement is completely solidified. This can be also applied to bone cement augmentation of the pedicle screws to prevent the cement leakage.

In conclusion, we described a unique case of intradural cement leakage after VP and review of the related literature. When neurological deficits occur after VP, a CT scan should be taken to confirm the pattern of cement leakage. In case of intradural cement leakage, surgical decompression should be recommended to improve neurological deficits. To prevent intradural cement leakage during the VP, the needle tip should not perforate the medial wall of the pedicle and cement of appropriate viscosity should be used.

## Data Availability

All data concerning the case are presented in the manuscript.

## Abbreviations

VP: vertebroplasty
ESR: erythrocyte sedimentation rate
CRP: C-reactive protein
Hb: hemoglobin
ALT: alanine aminotransferase
MRI: magnetic resonance imaging
CT: computed tomography

## References

[1] Teng MM, Cheng H, Ho DM, Chang CY (2006). Intraspinal leakage of bone cement after vertebroplasty: a report of 3 cases. AJNR Am J Neuroradiol.

[2] Jang HD, Kim EH, Lee JC, Choi SW, Kim K, Shin BJ (2020). Current Concepts in the Management of Osteoporotic Vertebral Fractures: A Narrative Review. Asian Spine J.

[3] Choi SH, Kim DY, Koo JW, Lee SG, Jeong SY, Kang CN (2020). Incidence and Management Trends of Osteoporotic Vertebral Compression Fractures in South Korea: A Nationwide Population-Based Study. Asian Spine J.

[4] Tamayo-Orozco J, Arzac-Palumbo P, Peón-Vidales H, Mota-Bolfeta R, Fuentes F (1997). Vertebral fractures associated with osteoporosis: patient management. Am J Med.

[5] Predey TA, Sewall LE, Smith SJ (2002). Percutaneous vertebroplasty: new treatment for vertebral compression fractures. Am Fam Physician.

[6] Lin BJ, Li CC, Ma HI (2015). Intradural cement leakage after percutaneous vertebroplasty. Turk Neurosurg.

[7] Hadjipavlou AG, Tzermiadianos MN, Katonis PG, Szpalski M (2005). Percutaneous vertebroplasty and balloon kyphoplasty for the treatment of osteoporotic vertebral compression fractures and osteolytic tumours. J Bone Joint Surg Br.

[8] Pateder DB, Khanna AJ, Lieberman IH. Vertebroplasty and kyphoplasty for the management of osteoporotic vertebral compression fractures. Orthop Clin North Am. 2007;38:409 – 18; abstract vii. 10.1016/j.ocl.2007.03.010 17629988

[9] Jay B, Ahn SH (2013). Vertebroplasty Semin Intervent Radiol.

[10] Chen YJ, Tan TS, Chen WH, Chen CC, Lee TS (2006). Intradural cement leakage: a devastatingly rare complication of vertebroplasty. Spine (Phila Pa 1976).

[11] Lee BJ, Lee SR, Yoo TY (2002). Paraplegia as a complication of percutaneous vertebroplasty with polymethylmethacrylate: a case report. Spine (Phila Pa 1976).

[12] Shapiro S, Abel T, Purvines S (2003). Surgical removal of epidural and intradural polymethylmethacrylate extravasation complicating percutaneous vertebroplasty for an osteoporotic lumbar compression fracture. Case report. J Neurosurg.

[13] Sabuncuoğlu H, Dinçer D, Güçlü B, Erdoğan E, Hatipoğlu HG, Ozdoğan S (2008). Intradural cement leakage: a rare complication of percutaneous vertebroplasty. Acta Neurochir (Wien).

[14] Kulkarni AG, Shah SP, Deopujari CE (2013). Epidural and intradural cement leakage following percutaneous vertebroplasty: a case report. J Orthop Surg (Hong Kong).

[15] Grelat M, Le Van T, Fahed E, Beaurain J, Madkouri R (2018). Rare complication of percutaneous technique: intradural cement leakage and its surgical treatment. World Neurosurg.

[16] Deramond H, Depriester C, Galibert P, Le Gars D (1998). Percutaneous vertebroplasty with polymethylmethacrylate. Technique, indications, and results. Radiol Clin North Am.

[17] Nguyen DH, Vu DD, Doan TN, Vo HL (2020). Safety of Balloon Kyphoplasty in the Treatment of Thoracic Osteoporotic Vertebral Compression Fractures in Vietnamese Patients. Clin Orthop Surg.

[18] Kim JH, Ahn DK, Shin WS, Kim MJ, Lee HY, Go YR (2020). Clinical Effects and Complications of Pedicle Screw Augmentation with Bone Cement: Comparison of Fenestrated Screw Augmentation and Vertebroplasty Augmentation. Clin Orthop Surg.

[19] Clark W, Bird P, Gonski P, Diamond TH, Smerdely P, McNeil HP (2016). Safety and efficacy of vertebroplasty for acute painful osteoporotic fractures (VAPOUR): a multicentre, randomised, double-blind, placebo-controlled trial. Lancet.

[20] Kallmes DF, Comstock BA, Heagerty PJ, Turner JA, Wilson DJ, Diamond TH (2009). A randomized trial of vertebroplasty for osteoporotic spinal fractures. N Engl J Med.

[21] Buchbinder R, Osborne RH, Ebeling PR, Wark JD, Mitchell P, Wriedt C (2009). A randomized trial of vertebroplasty for painful osteoporotic vertebral fractures. N Engl J Med.

[22] Klazen CA, Lohle PN, de Vries J, Jansen FH, Tielbeek AV, Blonk MC (2010). Vertebroplasty versus conservative treatment in acute osteoporotic vertebral compression fractures (Vertos II): an open-label randomised trial. Lancet.

[23] Hulme PA, Krebs J, Ferguson SJ, Berlemann U (2006). Vertebroplasty and kyphoplasty: a systematic review of 69 clinical studies. Spine (Phila Pa 1976).

[24] Lee MJ, Dumonski M, Cahill P, Stanley T, Park D, Singh K (2009). Percutaneous treatment of vertebral compression fractures: a meta-analysis of complications. Spine (Phila Pa 1976).

[25] Cortet B, Cotten A, Boutry N, Flipo RM, Duquesnoy B, Chastanet P (1999). Percutaneous vertebroplasty in the treatment of osteoporotic vertebral compression fractures: an open prospective study. J Rheumatol.

[26] Patel AA, Vaccaro AR, Martyak GG, Harrop JS, Albert TJ, Ludwig SC (2007). Neurologic deficit following percutaneous vertebral stabilization. Spine (Phila Pa 1976).

[27] Schmidt R, Cakir B, Mattes T, Wegener M, Puhl W, Richter M (2005). Cement leakage during vertebroplasty: an underestimated problem?. Eur Spine J.

[28] Lai PL, Tai CL, Chen LH, Nien NY (2011). Cement leakage causes potential thermal injury in vertebroplasty. BMC Musculoskelet Disord.

[29] Laredo JD, Hamze B (2005). Complications of percutaneous vertebroplasty and their prevention. Semin Ultrasound CT MR.

[30] Tang S, Fu W, Zhang H, Zhang H, Liang B (2019). Efficacy and safety of high-viscosity bone cement vertebroplasty in treatment of osteoporotic vertebral compression fractures with intravertebral cleft. World Neurosurg.

